# Advances in cell-based biosensors: Transforming food flavor evaluation with novel approaches

**DOI:** 10.1016/j.fochx.2025.102336

**Published:** 2025-03-01

**Authors:** Mahmoud Said Rashed, Esraa A. Abdelkarim, Tamer Elsamahy, Mabrouk Sobhy, Hany S. El-Mesery, Ali Salem

**Affiliations:** a Food Science and Technology Department, Faculty of Agriculture, Alexandria University, Alexandria 21545, Egypt; b Food Control Department, Faculty of Veterinary Medicine, Zagazig University, Zagazig 44519, Egypt; c Independent researcher, Zhenjiang 212013, China; d School of Agricultural Engineering, Jiangsu University, Zhenjiang 212013, China; e School of Food and Biological Engineering, Jiangsu University, Zhenjiang 212013, China; f School of Energy and Power Engineering, Jiangsu University, Zhenjiang 212013, China; g Agricultural Engineering Research Institute, Agricultural Research Center, Dokki, 12611 Giza, Egypt; h Civil Engineering Department, Faculty of Engineering, Minia University, Minia 61111, Egypt; i Structural Diagnostics and Analysis Research Group, Faculty of Engineering and Information Technology, University of Pecs, Hungary

**Keywords:** Flavor, Consumer acceptance, Cell sensors, Transducers, Cell immobilization

## Abstract

Food flavor, a blend of taste and smell, is key to consumer acceptance and food quality. Traditional sensory and instrumental methods often fail to replicate human sensory responses. This review discusses the role of cell-based biosensors in flavor evaluation, showcasing their sensitivity, specificity, and rapid response. Using living cells like taste and olfactory cells, these biosensors surpass traditional approaches. Advancements include microelectrode array systems with taste receptor cells for real-time detection of bitter, sweet, and umami substances and improved cell immobilization technologies for detecting complex odorant profiles. Challenges such as signal stability, selective detection, cell cultivation, and scalability persist. However, integrating artificial intelligence and portable technologies could broaden their applications. With the potential to revolutionize sensory analysis, cell-based biosensors offer a sustainable, precise, and scalable approach to food flavor evaluation, bridging sensory perception with advanced analytical methods and driving innovation in food science.

## Highlights

• Cell-based biosensors revolutionize food flavor evaluation with high precision.
• Utilize living cells for real-time taste and odour detection with high specificity.
• Overcome limitations of traditional sensory methods with enhanced reproducibility.
• Challenges include signal stability, scalability, and selective detection issues.
• Future lies in AI integration for portable, scalable, and precise flavor analysis.

## 1 Introduction

Consumer’ expectations of food quality have changed as people's standard of living has risen, going beyond the fulfillment of their nutritional needs. The flavor of food is one of the most important determinants of food quality and an essential characteristic for distinguishing between different foods (Heide & Olsen, 2018; Nag et al., 2019). The sensory evaluation of food plays a crucial role in quality control, product development and for the consumer. It serves as an important decision-making tool for the food industry by guiding product development and marketing strategies (Kumar et al., 2024; Lawless & Heymann, 2010). Flavor is one of the key drivers of consumer purchasing decisions, as shown by studies demonstrating a strong correlation between satisfaction and repeat purchases (Kumalasari, 2023; Lintong et al., 2023). Flavor can also attract consumers in the purchase phase, while its influence decreases in the consumption phase. For example, it was found that the aroma of bread and pastries in the store is an important point of attraction, but has less influence on the actual purchase decision (Zelený et al., 2023). The interaction of flavors and food ingredients is crucial for product acceptance, especially for fortified foods. Consumers are increasingly looking for products that offer a balance between health benefits and taste satisfaction. The interplay of flavors and food ingredients is critical to product acceptance, especially for fortified foods. Consumers are increasingly looking for products that offer a balance between health benefits and flavor satisfaction. Understanding flavor behavior can improve taste perception and thus influence consumer choices in health-oriented food markets (Chen et al., 2023).

Traditional sensory evaluation methods, primarily relying on trained human panels, have been the cornerstone of food flavor evaluation for decades and provide valuable insights into the organoleptic properties of foods. These methods can be broadly categorized into three main categories: discriminative, descriptive and affective tests to ensure reliable and valid sensory evaluations and assure product quality (Kumar et al., 2024). The aim of discriminative tests is to detect differences between samples. These include methods such as the triangle test, the duo-trio test and the paired comparison test (Lawless & Heymann, 2010). Such tests are primarily used for quality control and to detect minor changes in product formulations. Descriptive tests are quite different as they focus on a systematic sensory profile of foods and provide details on attributes such as appearance, aroma, flavor and texture. Common objective descriptive methods, including Quantitative Descriptive Analysis (QDA) and the Spectrum method, work with trained panellists who rate the intensity of certain attributes. Affective tests, often referred to as consumer tests, measure consumer acceptance, preference and liking for products. These include hedonic scaling, preference ranking and acceptance testing all rich sources of information for potential market success. Newer rapid methods such as Check-All-That-Apply (CATA), Napping and Temporal Dominance of Sensations (TDS) have also emerged alongside traditional methods to provide faster and more holistic approaches to sensory evaluation (Ruiz-Capillas & Herrero, 2021).

However, conventional approaches to sensory evaluation have their limitations. These include tester fatigue, subjective bias, high operating costs and time-consuming training requirements. In addition, the increasing demand for rapid, high-throughput screening in food development has made it clear that conventional methods do not meet the needs of modern industry (Wilson & Baietto, 2009). Furthermore, the complex nature of flavor perception, which involves multiple sensory modalities and chemical interactions, requires reliable and objective evaluation methods to ensure consistent product quality and meet consumer expectations. Standard analytical techniques such as GC, HPLC, LC-MS, GC-olfactometery, etc. are common technologies for the detection of target flavor components in food. Although these technologies allow the quantitative detection of various target flavor components in food, they cannot provide information on the taste and smell of the substances and cannot provide a comprehensive evaluation of taste, concentration and strength (Coulthard et al., 2022). Thereby these methods, in combination with other techniques such as sensory descriptive analysis, provide an understanding of the key attributes for consumer satisfaction and market success. The approach known as artificial sensory evaluation combines the sensory evaluation of the human body with the analysis of instruments to provide a more comprehensive and reliable evaluation result (Tao et al., 2021).

Artificial sensory evaluation also refers to the use of some other instruments, including the electronic nose, the electronic tongue and the machine vision system. By simulating the functions of the sense of smell, taste and sight, the characteristic signal of the target flavor is recognized based on multiple sensors and combined with the pattern recognition method to perform sensory evaluation. Compared with conventional sensory evaluation, intelligent sensory evaluation reduces the training burden on personnel and avoids the influence of subjectivity and other factors, but this method cannot replace the response of the taste system (from the tongue to the brain) to the comprehensive information of flavoring substances and the construction of sensitive materials for sensors. The adsorption and catalysis of special ions have a great effect on their ability to analyse and recognize the target substances (Ghasemi-Varnamkhasti et al., 2010). Intelligent sensory evaluation uses big data analysis to evaluate the flavor of food based on odour and taste information vectors.

The evaluation of food flavor using biosensors has become one of the research hotspots in recent years (Wang et al., 2020; Zhang et al., 2020). Enzyme sensors based on enzyme-catalysed redox reactions have been gradually used for the detection of taste compounds, with a focus on the analysis of umami and sweetness (Jayanthi Kalaivani & Suja, 2019; Sun et al., 2016). Compared with enzyme sensors, biosensors with taste tissues and receptors (Fan et al., 2022; Li et al., 2021; Xiao et al., 2019), as sensitive materials, can obtain sensitive components from nature and have a relatively long lifespan. The development and use of taste bud tissues have made the application of biosensors in flavor analysis more diverse (Lasconi et al., 2019).

Biosensors based on different olfactory cells have been developed for the evaluation and analysis of food flavors. These biosensors use flavor receptor proteins or whole cells as biosensing elements equipped with various electrochemical transducers (Kumar et al., 2021). It has also been proposed to use the chemosensory cells of the intestinal secretin tumor cell line (STC-1) as a taste recognition element, as their taste-specific cellular response can be decoded by multivariate data processing (García Lozano et al., 2019). These biosensors offer a non-invasive approach for taste recognition tests and can also be used to study taste transduction mechanisms in vitro (Chen et al., 2021). The development of microbial sensors using microbial immobilization technology has also enabled the sensitive determination of ethanol and the detection of compounds such as caffeine and mycotoxins (Zabadaj et al., 2019). Overall, biosensors based on taste and olfactory cells are promising in the field of food flavor assessment and offer potential for future research. In this review, we provide a general overview of recent advances and developments in taste and olfactory cell-based biosensors for food flavor assessment.

## 2 Cell biosensors

Cell biosensors use biological living cells as the sensitive elements of the sensor to identify target analytes and obtain characteristic information about them. The signal response characteristics of the biosensors exhibit a systematic relationship with the target analytes, allowing for effective evaluation (Hang et al., 2021). This relationship is influenced by several factors, including the design of the biosensor, the nature of bioelement and the transduction mechanism. Understanding these aspects can improve the reliability and sensitivity of biosensors in detecting certain analytes. According to Priya (2024), successful biosensors must maintain a linear response across different analyte concentrations to ensure accurate quantification. While a uniform signal response is beneficial, it is important to note that external factors such as temperature and pH can also affect biosensor performance, which can lead to variability in results (Priya, 2024).

Biosensors combine a biological component with a physicochemical detector, such as optical or electrochemical methods, to measure and quantify the interaction between the analyte and the biological element (Chalklen et al., 2020). These biosensors offer rapid and cost-effective detection with excellent sensitivity and specificity, making them valuable in various fields such as medicine, food safety, environmental monitoring, as well as in scientific research and day-to-day applications (Saffioti et al., 2020). The development of mechanical and electrical biosensors has provided real-time data with good temporal sensitivity and offers opportunities for advances in biosensing (Asal et al., 2019). In addition, the development of micro- and nanostructured biomaterials has enabled the creation of responsive biointerfaces that allow the readout of mechanics, biochemistry and electrical activity in real time and enable the observation of cellular processes with molecular specificity (Ley et al., 2018).

**Fig. 1:**
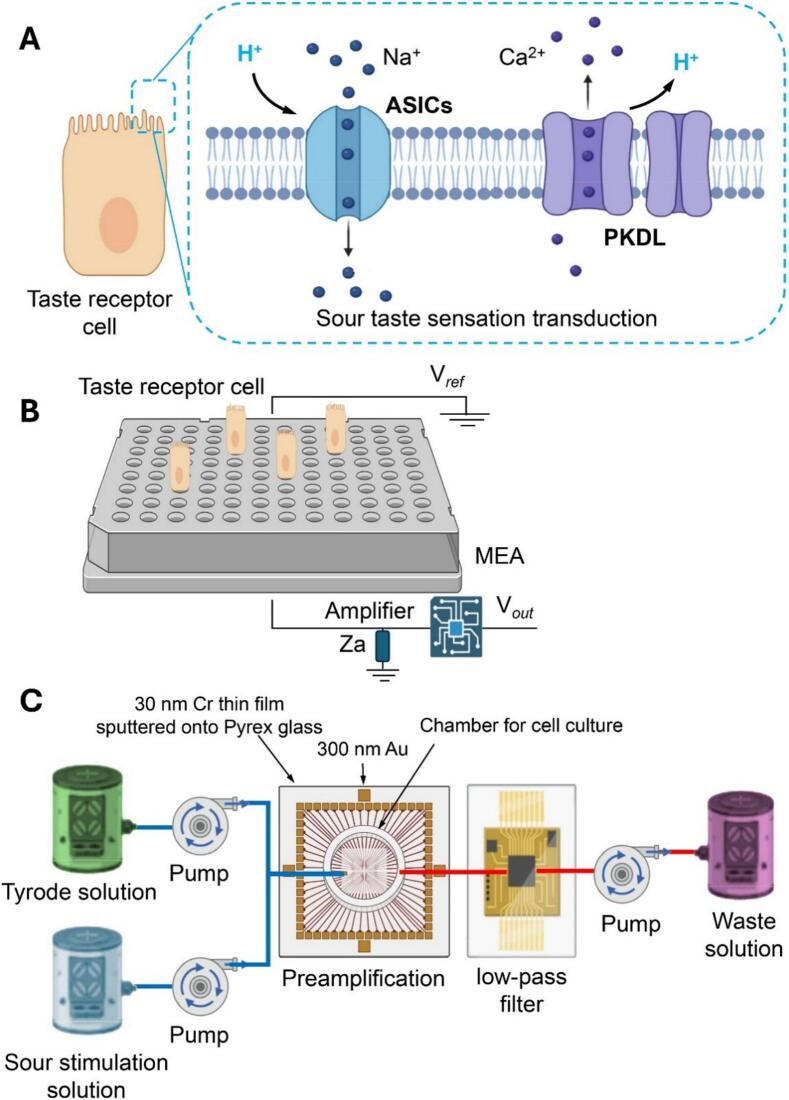
Sour taste transduction and bioelectronic tongue measurement. Proposed mechanisms of sour taste transduction involving ASICs and PKDL channels (**A**). Schematic representation of the acid-sensing biosensor (**B**). Bioelectronic tongue system for extracellular electrophysiological recordings from taste receptor cells (**C**). Adapted from Zhang et al. (2017).

Cell biosensor systems consist of three main components: a sensitive element (first-level receptor), a transducer and a signal processing system. These systems can be categorized into microbial cell sensors, which use bacteria, fungi, yeasts and algae as sensitive elements, and animal cell sensors, which use higher eukaryotic cells such as fish, rats and human cells as sensitive elements (Michaelis & Wegener, 2020; Saffioti et al., 2020). The use of microbial cell sensors enables non-invasive and label-free examination of cells over long observation periods, making them suitable for various applications such as drug testing and risk assessment (Michaelis & Wegener, 2020). On the other hand, animal cell sensors offer excellent sensitivity and specificity for a variety of purposes, from scientific research to day-to-day applications (Asal et al., 2019). Both types of cell sensors are valuable tools for studying cell behavior and detecting biologically active molecules (Jeon et al., 2018). With the continuous development of cell biosensors, their applications in environmental monitoring, medical diagnosis, pharmaceuticals, food analysis, etc. are becoming more and more advanced (Gupta et al., 2019). This article focuses on the research progress of cellular biosensors in the evaluation of taste and smell. Fig. 1 shows a typical structure of a cellular biosensor and its application in the analysis of different flavor substances. Progress of cellular biosensors in the detection of flavors.

Studies have shown that other cells besides taste cells can also recognize flavor compounds, so that the detection of taste substances based on cell biosensors has developed rapidly in recent decades (Li, 2005; Wu et al., 2017). Cell biosensors used for the detection of taste substances are mainly divided into taste cell sensors, mouse sperm cell sensors, intestinal cell sensors, and cardiomyocyte sensors according to the different sensitive components. For example, Zhang et al. (2020) found that one of the key research components of the gustatory system is taste biosensors based on mimicking or natural mammalian taste components. Also, the light-addressable potentiometric sensor (LAPS) is a versatile platform for chemical and biological sensing as well as the ability to measure various ions or molecules (Yoshinobu & Schöning, 2021). In addition, optical biosensors, including synthetic dyes and genetically encoded proteins, have been used to investigate taste-related intra- and intercellular signaling mechanisms (von Molitor et al., 2020). In addition, chemosensory cells of STC-1 were used for taste recognition, which represents a new approach for non-invasive taste sensing assays (Zabadaj et al., 2019).

### 2.1 Taste cell-based biosensors

**Fig. 2:**
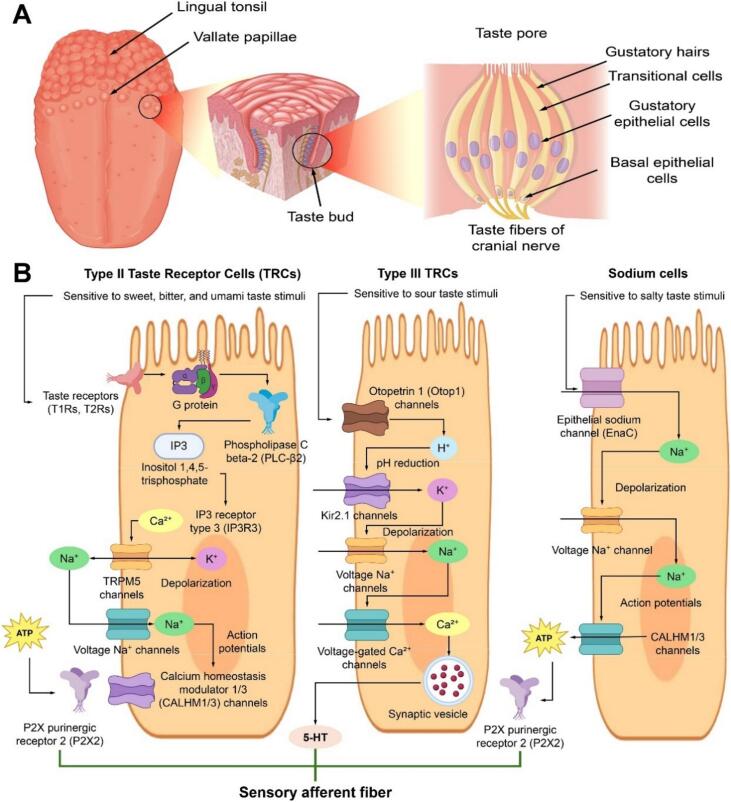
Human taste perception: Structure and transduction. Anatomical distribution and structural organization of lingual papillae, taste buds, and taste receptor cells (**A**). Schematic illustration of taste signal transduction pathways in various taste receptor cell types in response to bitter, umami, sweet, sour, and salty stimuli (**B**). Adapted from Y. Li et al. (2023).

Different research studies have used various taste systems to identify molecules in potential foods. According to ultrastructural characteristics, gene expression patterns, and cell functions, taste cells can be divided into three types, namely type I cells, type II cells, and type III cells, and their structures are shown in Fig. 2 (Li et al., 2023). Sweetness, bitterness, and umami are all sensed by type II taste cells (Lee & Owyang, 2019), in which taste receptors T1R2 and T1R3 combine to form sweet taste receptors, T2Rs are bitter taste receptors, and T1R1 and T1R3 combine to form umami taste receptors. Type III cells perceive sour stimuli (Matsumoto et al., 2019), whereas the taste cells that perceive salty stimuli (NaCl) have not been identified (Chandrashekar et al., 2010). For example, ionotropic receptors are responsible for the recognition of salt and sour, while G protein-coupled receptors (GPCR) pathways get activated by bitter, sweet and umami tastes. Banik and Medler (2021) found that both GPCRs and a common PLCβ2/IP3R3/TRPM5 signaling pathway are involved in the transduction of bitter, sweet and umami stimuli by Type II taste cells. Several signaling pathways, including PLCβ2 and PLCβ3, are critical for taste transduction. Loss of these signaling pathways leads to diminished, but not abolished, taste responses (Banik et al., 2019). The TRPM4 and TRPM5 channels are also important. Their absence leads to significant impairments in taste recognition, but some residual responses remain (Banik et al., 2018). According to numerous studies, the absence of particular signaling elements, particularly Type II taste cells, reduces but does not completely eradicate taste reactions to bitter, sweet and umami stimuli (Banik et al., 2019). This is confirmed by the work of Larson et al. (2020), who found that in the absence of Type II cells, as in Skn-1a knockout mice, Type III cells can still respond to these stimuli, albeit with reduced efficacy. This suggests that Type III cells also contribute to taste perception even in the absence of Type II cells. Moreover, recent research has shown that there is a group of taste cells distinct from Type II cells that exhibit broad responsiveness to a variety of flavor attributes such as bitter, sweet and umami (Banik et al., 2020; Banik & Medler, 2021). These results indicate that the signaling processes in taste cells are more complex than previously appreciated (Banik et al., 2020; Kinnamon & Finger, 2019). Therefore, the choice of taste cells as sensitive elements of biosensors is one of the most effective ways to express taste signals (Ervina et al., 2021).

Taste cells are commonly used as biosensor elements of taste biosensors. As a cell source for sensitive elements, they have the advantage that they can detect signals sensitively and transmit them precisely (Wang et al., 2020). The cultivation of primary taste cells derived taste buds is challenging due to their high microenvironmental requirements and their limited ability to survive and proliferate in vitro (Guarascio et al., 2021). Therefore, the use of taste stem cells to generate organoids offers a more viable approach to study taste cell function and taste perception (Shechtman et al., 2021). Taste stem cells can be used to grow organoids which can differentiate into taste cells in vitro (Rodriguez et al., 2021). They can be characterized as a sensitive element, and intact taste cells can be used as a basic unit for taste perception (Guarascio et al., 2021). Thus, real taste and intracellular signal transduction can be highly simulated in vitro and stimulation by taste substances can be perceived more directly, which is the basis for the construction of taste cell sensors (Wu et al., 2014). However, the cultivation of organoids is also difficult. At the same time, there are problems such as the difficulty of controlling the types of cultured taste cells, the small proportion of taste cells in organoids, and the short survival time, which limits the development of taste cell sensors to some extent (Wu et al., 2017).

In the construction of the sensor, the researchers used various secondary receptors to record the extracellular potential of the neurons. These include field-effect transistors (FETs) (Guo et al., 2020), Microelectrode arrays (MEAs) (Tanwar et al., 2022), LAPS and glassy carbon electrodes (GCEs). FET arrays have been used to record intracellular potentials of single cells (López-Villanueva & Rivadeneyra, 2020). MEA detects changes in current, impedance and potential due to the release of target molecules and movement through the extracellular space (Tedjo et al., 2016).

LAPS devices use the field effect to convert electrophysiological activity into regional charge carrier redistribution, enabling spatially resolved photocurrent recordings (Stumpf & Hoffmann, 2016). GCEs have been used as capacitive sensors for various applications. These receptors offer various possibilities for recording neural activity, such as spatial resolution and label-free imaging. Among them, MEA and FET are suitable for long-term detection, but their recording sites are limited to electrode or gate sites.

Researchers have explored a range of receptors to enable the recording of extracellular potentials from neurons. One approach is to simulate extracellular recordings using neuron models with multiple active or passive compartments (Tran et al., 2018). Another approach is to use electrical extracellular recordings which can provide information about the firing of action potentials and the way neurons integrate synaptic inputs (Lindén et al., 2014). Nanocavity sensor arrays have also been developed for parallel extracellular recordings and offer high spatial resolution and low electrode impedances (Czeschik et al., 2014). In addition, a portable wireless telemetry system for recording extracellular neuronal activity has been developed that offers the flexibility to select different channels (Heredia-López et al., 2009). Finally, a novel platform combining a high-density microelectrode array with a poly (dimethylsiloxane) channel device has been developed to study axonal physiology and information processing (Lewandowska et al., 2015). Each of these approaches has its own advantages and potential applications for recording extracellular potentials of neurons. Table 1 summarizes the application of taste cell-based sensors in the evaluation of taste substances in recent years.

**Table 1:** Research and applications of sensors based on taste cells.

Sensitive components	Secondary sensor/detection method	Application	Detection range/(mol/L)	References
Rat taste epithelium	MEA	Rapid identification of different bitter substances	Quinine: (1.0–100.0) × 10^−6^ Denatonium benzoate: (1.0–10.0) × 10^−5^ Cycloheximide: (1.0–10.0) × 10^−5^	(Liu, Zhang, Zhang, Zhao, et al., 2013)
	MEA	Identification and detection of salt (NaCl)	(1.0–5.0) × 10^−4^	(Liu, Zhang, Zhang, Hu, et al., 2013)
	MEA	Evaluate natural and artificial sweeteners	Glucose: (5–15) × 10^−5^ Saccharin: (5–15) × 10^−6^	(Liu & Yan, 2012)
rat taste cells	GCE	Can be used to more precisely elucidate the mechanism of interaction of an irritant substance with its receptor	Gingerol solution: (1.0–9.0) × 10^−16^ Capsaicin: (1.0–10.0) × 10^−16^	(Qiao et al., 2015)
pig tongue epithelial cells	GCE	Kinetic analysis of receptors for bitter substances (sucrose octaacetate, denatonium benzoate, and quercetin)	Sucrose octaacetate: (1.0–10) × 10^−17^ Denatonium benzoate: (1.0–10,000) × 10^−16^ Quercetin: (0.01–1000) × 10^−15^	(Wei et al., 2017)
Rat taste bud cells	GCE	Study on the Antagonism Kinetics of 6 Different Substances to Capsaicin	NM⁎	(Xiao et al., 2019)
	LAPS	Documenting Bitter Taste Signaling	NM	(Du et al., 2018)
rat taste receptor cells	LAPS	Realize the perception of sour taste	NM	(Chen et al., 2009)

**MEA**; Microelectrode Array; **GCE**; Glassy Carbon Electrodes; **LAPS**; light-addressable potentiometric sensors.

⁎ NM: Not Mentioned.

Taste cell sensors have the advantage of having different types of detection and high sensitivity, but they also have disadvantages, such as difficulty in obtaining sensitive components, low stability, and difficulty in mass extraction. These sensors have been used to evaluate various sweet, spicy, bitter and sour substances (Hao et al., 2023; Kwon et al., 2017; Zabadaj et al., 2019; D. Zhang & Liu, 2015). However, there is still a need for taste sensors with higher sensitivity to meet the increasing expectations of the public (Kwon et al., 2017). Moreover, the sensitivity and selectivity of taste sensors based on electronic tongues are lower than those of biological taste sensations (Zhang & Liu, 2015). Therefore, it is more reasonable to develop a cell sensor as a biosensor model, which has better sensitivity and selectivity in the basic theoretical research of the taste system (Qiao et al., 2015; Xiao et al., 2019), rather than using it as a tool to analyse flavor compounds.

### 2.2 Biosensor based on mouse sperm cells

Studies have shown that orthologous bitter taste receptor gene pairs are found in both human and mouse genomes, and Xu et al. (2013) have confirmed that bitter taste receptor genes are abundantly expressed in mouse testis tissue. Bitter taste receptor genes have been preserved throughout evolution and are capable of recognizing a variety of bitter chemicals (Governini et al., 2020; Lossow et al., 2016). Mouse sperm cells were used as sensitive materials in an impedance sensor to specifically detect bitter substances. The optimal cell density for the sensor was set at 106 cells/mL in 4 h. The detection ranges for phenylthiourea and quinine were 10 to 200 μmol/L and 62.5–1000 μmol/L, respectively, with detection limits of 4 μmol/L and 40 μmol/L. In addition, there was a linear correlation between the increase in sensor impedance value and the concentration of bitter substances (Tian et al., 2020). This method combines the specific response of mouse sperm cells to bitter substances with the characteristics of cell impedance sensors, which can measure the physiological and ecological changes of cells in real time and without invasion, and provides a new idea for the detection of bitter substances. Hui et al. (2014) used the principle of cell impedance to construct a biosensor for bitter taste based on mouse sperm cells, investigated the cell impedance response to four bitter compounds, and explored the feasibility of the cell sensor to detect other basic tastes. The results show that the cell sensor is only specific to bitter taste and shows no signal response to other taste substances, which provides a new idea for the specific detection of bitter taste in mixed taste substances. Tian et al. (2020) constructed a new biosensor based on sperm cells. The sensor uses live mouse sperm cells as the main sensitive element, utilizes an intracellular calcium ion fluorescent probe (Fluo4-AM) as a sensor, and combines flow cytometry to achieve rapid quantitative detection of bitter compounds. The fabricated biosensor enabled a preliminary classification of three bitter substances using principal component analysis (PCA). The produced biosensor was practical, inexpensive and easy to handle and can respond to bitter compounds in a dose-dependent manner with high sensitivity, high specificity and a low detection limit. The development of new detection methods to increase the sensing speed of sperm cell sensors is a breakthrough for their commercial application. Currently, sperm cell sensors are mainly used for bitter taste detection, but their application range is limited. The sensors are characterized by a simple method, low price, high sensitivity, strong specificity and low detection limit. However, if the cell impedance principle is used for detection, the sensor responds slowly to external taste stimuli, which makes it difficult to identify the flavoring substances quickly. Therefore, there is a need for new methods that can improve the sensing speed of sperm cell sensors (Hidayatullah et al., 2021; X. Wei et al., 2021).

### 2.3 Intestinal cell-based biosensors

In recent years, more and more researchers have elucidated the expression mechanism of taste receptors in intestinal cells and found that sweet, bitter and umami receptors are expressed in the intestines of mice, rats and humans. Bitter substances are recognized in the human colon by the bitter taste receptors T2Rs, including T2R3, T2R4, T2R5, T2R10, T2R13, T2R38, T2R39, T2R40, T2R43, T2R44, T2R435, T2R46, T2R47, T2R49, T2R50 and T2R60. Umami substances can be recognized by T1R1 and T1R3 heterodimers (Kim et al., 2015; Yoshida et al., 2019), and sweet substances can be recognized by T1R2 and T1R3 heterodimers (Chéron et al., 2019; Daly et al., 2021; Karl et al., 2020). Due to the properties of intestinal cells expressing taste receptors, sensors using them as sensitive materials have also started to attract attention. It has been reported that NCI-H716 and STC-1 cells are mostly used as sensitive elements of intestinal cells to construct impedance sensors. Most results show that the two cell types enable the selective recognition of bitter and sweet substances (Liu, Zhang, Zhang, Zhao, et al., 2013; Wang, Carey, et al., 2010); and they can be quantitatively detected (Hui et al., 2012). Hui et al. (2014) cultured NCI-H716 and STC-1 cells on screen-printed carbon electrodes to construct two types of cell sensors. The results showed that the NCI-H716 cell sensor could discriminate 13 sweetener mixtures and 7 sweeteners. In the tastant mixture of sucrose, the detection range of sucrose is 0.71 × 10^−1^–2 × 10^−1^ mol/L; the detection range is 2.6 × 10^−5^–5 × 10^−5^ mol/L. This type of sensor is relatively easy to construct by culturing specific intestinal cells as sensitive elements, and has attracted attention for its rapid response, high sensitivity and strong selectivity (Dyer et al., 2005; Prandi et al., 2013). However, the current research only evaluates some sweet and bitter substances, and the detection conditions need to be optimized before analysis and detection, such as signal amplification, feature extraction, etc.; in addition, some biological methods need to be used to verify whether the signal is mediated by the binding of tastants to selective GPCR. In the future, it is necessary to further investigate the recognition mechanism and expand the scope of application.

### 2.4 Biosensors based on cardiomyocytes

According to Foster et al. (2013), various studies have shown that taste receptors are not only expressed in the taste system, but also in rat cardiomyocytes. The ubiquitous expression of taste receptors offers multiple possibilities for the construction of biosensors. Wei et al. (2019) used rat cardiomyocytes as a sensitive element and MEA as a secondary sensor for the first time to record the electrophysiological signals of cardiomyocytes in vitro and develop a bitter and umami taste detection system based on in vitro bionic cells. The results show that the cardiomyocytes adhere to the surface of the sensor and grow well. The syncytia they form conduct potential and beat mechanically, indicating good biocompatibility of the surface coating. The biospecificity of the taste components was verified and PCA was used to identify different taste components. Two bitter substances (denatonium benzoate and difenidol) and umami compounds (sodium glutamate) were successfully identified. Specific detection: the detection range of denatonium benzoate is 1.0 × 10^−5^–6.4× 10^−4^ mol/L, the detection limit is 3.46 × 10^−6^ mol/L, and the detection range of difenidol is 5.0× 10^−6^–3.2 × 10^−4^ mol/L, the detection limit is 2.92 × 10^−6^ mol/L, the detection range of sodium glutamate is 1.0 × 10^−6^–4.0 × 10^−3^ mol/L, the detection limit is 1.6 × 10^−6^ mol/L. The taste cell sensor based on cardiomyocytes and MEA is a new taste detection method with good specificity in the differentiation of bitter and umami compounds.

It should be noted that the exact mechanism of action of most GPCRs in the myocardium is not yet fully understood and will require further research in the future. GPCRs are widely distributed throughout the body, including the heart, and offer potential as alternative sensing elements for taste biosensors. Cardiomyocyte-based sensors have promising applications in taste recognition and drug research (Ahmad & Dalziel, 2020; Foster et al., 2015).

## 3 Research progress of cellular biosensors in the detection of odorants

Olfactory sensor technology can simulate the human olfactory organ to realize the evaluation of odour substances. In recent years, the research of olfactory cell- based biosensors has attracted great attention and has been applied in many fields such as biomedicine, food, medicine and environmental protection (Dung et al., 2018; Jiang & Liu, 2020; Son & Park, 2018). According to the different sources of cells used as sensitive elements, cellular biosensors for odorant detection are mainly divided into the following three categories: Vertebrate odorant cell sensors, insect receptor cell sensors, and heterologous cell sensors.

### 3.1 Biosensors based on vertebrate olfactory cells

The olfactory cells of vertebrates can detect various odorants with extremely high sensitivity and are increasingly seen by researchers as potential targets for the development of cellular sensors. The axons of the olfactory cells in the olfactory epithelium form the olfactory nerve, and the olfactory neurons have thousands of olfactory receptors which can sense stimulation from different types of odours. Therefore, the olfactory sensor based on vertebrate olfactory cells has the characteristics of strong specificity, high sensitivity and fast response, and is an ideal biomaterial for olfactory sensors (Cave et al., 2019). Liu et al. (2010) constructed a bioelectronic nose by immobilizing olfactory cells on the surface of LAPS. Stimulation tests were performed with acetic acid and diacetyl. The results showed that LAPS can detect changes in the extracellular potential of receptor cells in the olfactory epithelium and distinguish acetic acid and diacetyl by PCA. Such systems have the potential as olfactory biosensor neuron-on-chips for the detection of odorants such as acetic acid and diacetyl. However, the olfactory cells are usually fixed directly to the surface of the transducer, resulting in a random distribution of cells and uncontrollable coupling between cells and transducer. This obvious defect leads to various limitations in the performance and application of such bionic olfactory cell biosensors. Therefore, how to realize controllable and efficient coupling of olfactory cells and sensors is also one of the challenges in improving the performance of olfactory cell sensors. Du et al. (2015) have found a new solution to the problem of random cell distribution using the method of DNA-directed site-specific cell immobilization. In this method, rat olfactory cells are used as sensitive elements, MEA chips are used as sensors, the cells are covalently linked to single-stranded DNA on the plasma membrane, and complementary single-stranded DNA is printed on the surface of the support in a certain pattern, thereby controlling the fixation of the cells on the surface of the solid support to form the ideal cell pattern. The olfactory cell sensor is stimulated by octanal and acetaldehyde, and the change of membrane potential of the olfactory cell is effectively monitored, thereby monitoring the response of the sensor. This method not only realizes the controllable site-specific coupling of olfactory cells and MEA, but also allows the immobilized olfactory cells to form an ideal distribution on the sensor surface and respond to specific odour stimuli (octylaldehyde and acetaldehyde). The coupling efficiency can be greatly improved, and the stability of the olfactory cell sensor can be enhanced. Zheng et al. (2016) reviewed progress in combining microelectronics with olfactory cells, highlighting the potential for enhanced biosensor performance through nanotechnology and microelectronics. Based on this ssDNA with different base sequences can also be attached to different olfactory cells to construct chemical-sensing microcell arrays. For example, the work carried out by Lee et al. (2009) showed that electrical stimulation can enhance cellular activity and increase response signals in olfactory receptor-expressing cells.

Also, the use of nucleic acids as sensing elements in sensor arrays has been explored, with Pu et al. (2022) reviewing their potential for differentiating multiple targets. These advancements suggest the possibility of constructing chemical-sensing microcell arrays using ssDNA with different base sequences attached to olfactory cells. However, the signal response obtained by this kind of olfactory sensor is the superposition of electrical signals from multiple olfactory receptors, which may bring certain difficulties to the later data analysis.

### 3.2 Biosensors based on insect receptor cells

Scientists have identified the functional properties of more than 100 odorant receptors of various insect species such as fruit flies, mosquitoes, moths and beetles (Wicher & Miazzi, 2021; Yan et al., 2020). These insect odorant receptors selectively sense different types of odorants, including alcohols, aldehydes, ketones, acids, hydrocarbons and aromatics (Wang, Hui, & Deng, 2010). Termtanasombat et al. (2016) used insect cells (Sf21 cell line) as odorant sensing elements, converted the cellular responses into non-invasive ones, visualised the fluorescence intensity by calcium imaging, and adopted the odorant sensor pair (E, Z)-10,12-hexadecenol, *trans*-10 and *cis*-12-hexadecadienal. The results showed that the heterologous protein gene was stably integrated into the genome of the cell line and that the cells stably expressed insect odorant receptors and could selectively differentiate between different smells. Calcium imaging prevents mechanical damage to the cells and is able to respond rapidly within ∼13 s. This method not only overcomes the short lifespan of cell-based biosensors such as odour sensors that use live cells expressing insect olfactory receptors, as previously used by Misawa et al. (2010), but can also be maintained for at least 2 months, and stability is also greatly improved.

**Fig. 3:**
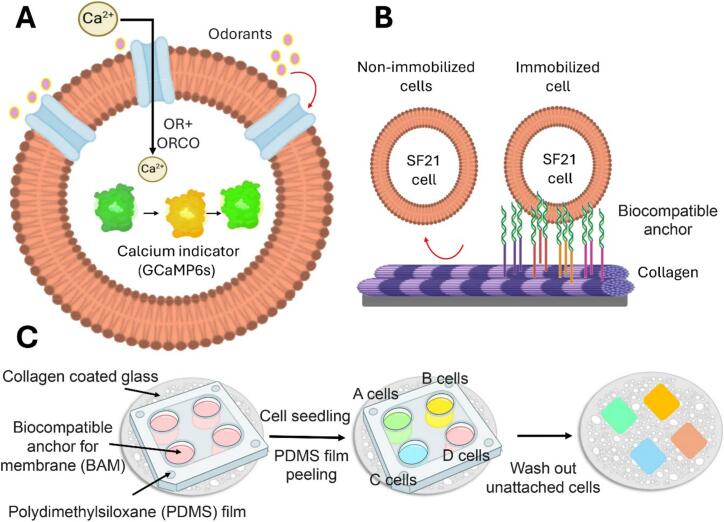
Development of a cell-based odour sensor array. Odorant detection mechanism in Sf21 cells expressing odorant receptor (OR) or co-receptor Orco, coupled with the genetically encoded GCaMP6s (**A**). Cell immobilization method utilizing a biocompatible membrane anchor (**B**). Multi-cell line array patterning procedure (**C**). Adapted from Termtanasombat et al. (2016).

Currently, although multiple odour-sensing cell lines expressing different olfactory receptors can be constructed, methods to integrate multiple cell lines into odour-sensing arrays have not yet been developed. Based on this assumption, Termtanasombat et al. (2016) used cell patterning technology to fabricate an odour sensor with an array pattern of multiple odour-sensing cell lines to combine multiple cell lines on the same surface (Fig. 3). The principle is also the use of Sf21, with the cell line acting as the odour sensing element and reflects the response to the target odour through fluorescence intensity. Array patterns expressing the cell lines Or13a, Or56a, BmOR1 and BmOR34 were successfully generated using patterned polydimethylsiloxane membrane templates and cell fixation reagents. The detection of 1-octen-3-ol, geosmin, (E,Z)-10,12-hexadecenol and *trans*-10, *cis*-12-hexadecadienal as odorants demonstrated the ability of the sensor to discriminate multiple target odorants.

Cell sensors based on such insect receptors have the advantages of good stability, a long available response time, the ability to express multiple cell line receptors, and the ability to discriminate a variety of odours, but there are still common problems as with other types of cell sensors. These include the complex and time-consuming pre-processing, the low efficiency of the final receptor expression, environmental conditions such as temperature and humidity that may affect the performance of the sensor, and the high cost of the final construction of the sensor, which limits the commercial application of this type of sensor.

### 3.3 Biosensors based on heterogeneous cells

In the past 10 years, many researchers have attempted to express olfactory receptor proteins in heterologous cell systems (Persaud, 2012). Some olfactory receptors are combined with other biological materials (such as cells) through artificial transduction systems, and physical and chemical detectors or transduction microsystems are used to express signals to achieve the purpose of smelling. Studies have shown that several receptor proteins can be expressed in *Escherichia coli* cells (Lee et al., 2018), yeast cells (Fukutani et al., 2011; Marrakchi et al., 2007), and *Xenopus laevis* melanocytes (Suska et al., 2009).

Table 2 summarizes the research progress of some heterologous cell-derived olfactory sensors. Compared to other types of olfactory sensors, heterologous cellular sensors can express only one desired type of olfactory receptors on the plasma membrane and they have better specificity because the natural structure of the olfactory receptors is preserved. Moreover, heterogeneous cellular sensors allow the transfer of markers, which facilitates the efficient immobilization of olfactory receptors (Fukutani et al., 2011; Lee et al., 2018; Suska et al., 2009). However, the study also revealed some problems in the construction of biosensors from heterologous cells, such as the fact that the production of olfactory receptors based on expression is time-consuming, labor-intensive, and relatively inefficient. In addition, the product of this method contains some unrelated proteins that require additional purification. There are also many difficulties in the functional analysis of the expressed olfactory receptor proteins, and how to implement the anti-apoptosis strategy of cells well is an important problem in the research of biosensors from heterologous cells (Ko & Park, 2005).

**Table 2:** Recent research progress on olfactory sensors based on heterologous cells.

Analytical method	Odour substances	Examination range	References
Human Olfactory Receptor 1A2 Nanodiscs (hOR1A2ND) Embedded and Functionalized on Floating Electrode Carbon Nanotube Field-Effect Transistors	Geraniol and Citronellol (rose scent)	Geraniol: 1 fmol/L Citronellol: 10 fmol/L	(Lee et al., 2018)
Expression of mouse trace amine-associated receptor 5 (TAAR5) in *Xenopus laevis* melanocytes	Amines such as trimethylamine	1 × 10^−6^–100 × 10^−6^	(Suska et al., 2009)
*Saccharomyces cerevisiae* cells expressing human olfactory receptors (OR 17–40) combined with conductance-staggered microelectrodes	Xinyang jasmonal	0.1 mol/L	(Marrakchi et al., 2007)
Production of Olfactory and Taste Receptors Immobilized in Multichannel Carbon Nanotube Field-Effect Transistors in *Escherichia coli* Cells	Octanol, Hexanal, Trimethylamine, and Thyroxine	ND	(Liu & Yan, 2012)
The olfactory receptor ODR-10 was transfected on the plasma membrane of neuroblastoma SH-SY5Y cells and cultured on the microelectrode	Diacetyl	1 × 10^−3^–1 × 10^−1^ mol/L	(Gao et al., 2019)

## 4 Challenges for the broad application of cell-based biosensors in the evaluation of food flavors

Cell-based biosensors offer promising potential for food flavor evaluation due to their specificity, sensitivity, and ability to mimic physiological environments (Wei et al., 2024; Ye et al., 2019).Despite their promising potential, cell-based biosensors face several significant limitations and constraints in food flavor evaluation applications.

1- The specificity and cross-reactivity of cellular responses pose a major challenge, as food matrices often contain complex mixtures of compounds that can affect sensor performance and generate false-positive signals. Cell-based biosensors often have problems with specificity when exposed to complex food matrices containing numerous compounds that can interfere with sensor responses (Mendoza et al., 2024). Cross-reactivity can lead to false-positive signals, making it difficult to accurately assess flavor profiles in foods (Liu et al., 2024). Optimizing response times is essential for practical applications but remains a challenge in the development of these biosensors. Mendoza et al. (2024) suggest that the integration of different detection elements and transducers can improve detection capabilities, but this requires careful design and testing.
2- The stability of biosensors is crucial, as fluctuations in environmental conditions can affect their performance. For example, temperature fluctuations, pH changes and matrix effects can affect the reliability and reproducibility of sensors (Mendoza et al., 2024). These results are also confirmed by Fetter et al. (2024), as electrochemical aptamer-based sensors show promising accuracy in physiological ranges of ionic strength, pH and cation composition, while temperature fluctuations can significantly affect their performance.
3- The high cost of development and maintenance, along with the necessity for specialized expertise and equipment, significantly hinders the widespread industrial adoption of advanced technologies. In addition, the lack of skilled personnel to implement and maintain advanced technologies, such as AI and mechatronics, poses a significant challenge (Lopez-Garcia & Rojas, 2024; Oke et al., 2023). Organizations often struggle to find or develop the necessary expertise, which can delay or prevent the adoption of innovative solutions (Laddha & Agrawal, 2024). Moreover, Technological skepticism and the complexity of integrating new systems into existing infrastructures are prevalent issues (Bagherian, Srivastav and Mukherjee, 2024a, Bagherian, Srivastav and Mukherjee, 2024b; Lopez-Garcia & Rojas, 2024). Furthermore, the need for robust IT infrastructure and ongoing adaptation to new technologies is critical for successful implementation (Bagherian, Srivastav and Mukherjee, 2024a, Bagherian, Srivastav and Mukherjee, 2024b).
4- The limited shelf-life of cell-based biosensors poses significant challenges for their long-term deployment, primarily due to the need for frequent recalibration and cell culture maintenance. According to Vermeulen et al. (2024) maintaining relative humidity below 50 % during storage is crucial to prevent dehydration and performance loss. Also, utilizing food packaging techniques, such as vacuum and modified atmosphere packaging, can significantly extend the shelf-life of immunosensors to over six months when stored under optimal conditions.
5- Standardization issues in laboratory protocols significantly impact the replicability and interpretation of results across different settings. In preclinical studies, harmonization of protocols across laboratories significantly reduced variability in results, demonstrating that aligned methodologies lead to more replicable findings. While harmonization is beneficial, introducing systematic variations in environmental factors (e.g., testing time, light intensity) did not further reduce variability, indicating that such factors may not be as influential as previously thought (Arroyo-Araujo et al., 2022). The need for standardized conditions is also critical in imaging protocols, where variations in hardware and settings can lead to inconsistent results across different platforms Malyarenko et al., 2023). Emerging technologies, such as advanced spectroscopic methods, face challenges in standardization due to inter-laboratory variations and the need for rigorous validation to ensure clinical applicability (Morais et al., 2019).
6- The scalability of biosensor production while ensuring consistent performance metrics remains a major challenge for commercial applications. Enzyme stability, integration complexity and high production costs are the main issues preventing widespread adoption. According to Mor (2024), enzymatic biosensors often suffer from enzyme denaturation under extreme conditions, which affects lifetime and performance. Variability in enzyme activity and immobilization techniques can also lead to inconsistencies and affect sensor reliability. Problems with integrating biosensors into existing industrial processes require compatibility with automated systems, making scalability difficult. Electrochemical biosensors face challenges with heterogeneous clinical samples, which require complex sample processing technologies and increase manufacturing costs (Akhlaghi et al., 2024). In addition, high production costs and regulatory barriers limit the scalability of biosensors (Andre et al., 2024). Innovations in materials science and AI technologies may offer ways to improve scalability and performance metrics (Li et al., 2024; Zhang et al., 2024).

## 5 Ethical concerns and regulatory compliance

The ethical concerns related to the use of human or animal cells in food testing applications are multifaceted, especially with regard to the authentication of the cell source and possible genetic modifications. These issues require careful consideration of the impact of the use of advanced biotechnologies such as CRISPR on food safety and authenticity. Ensuring the authenticity of food products, especially those derived from animal cells, is crucial to maintain consumer trust and safety (Vaithiyanathan et al., 2023). Advanced molecular techniques, including CRISPR-based assays, are employed to authenticate food sources, detecting specific nucleic acid components to prevent food fraud (Deng et al., 2024)(Vaithiyanathan et al., 2023).

Cell-based biosensors, particularly whole-cell microbial biosensors (WCMBs), face several regulatory compliance and safety challenges in the evaluation of food flavors. These challenges stem from the need for strict adherence to safety standards, biosensor stability and the complexity of food matrices. Regulatory agencies such as the FDA and EFSA set strict guidelines for the use of genetically modified organisms (GMOs) in food applications, which may limit the use of WCMBs (Gupta et al., 2024). Compliance with food safety standards is crucial as any non-compliance can lead to public health risks. This includes ensuring that biosensors do not introduce contaminants into food products (Omidvar & Farzad, 2024). The stability of microbial cells in biosensors is a major concern as environmental factors can affect their performance and reliability in detecting food flavors (Mendoza et al., 2024). Complex food matrices can interfere with biosensor readings, making accurate assessment of flavors difficult and requiring advanced signal processing techniques (Hu et al., 2024; Liu et al., 2024). Despite these challenges, the potential for innovative solutions, such as bio-automated systems and synthetic biology approaches, may enhance the effectiveness and compliance of cell-based biosensors in food flavor evaluation. However, the balance between innovation and regulatory adherence remains a critical consideration in their development.

## 6 Outlooks and future prospects

The future development of cell-based biosensors for the evaluation of food flavors offers exciting opportunities for revolutionary advances in sensor technology. The integration of CRISPR-Cas9 gene editing technology with cell-based biosensors holds great promise for the development of highly specific and customizable sensing elements for complex flavor compounds (Ansori et al., 2023). CRISPR-based biosensors utilize the collateral cleavage activities of Cas effectors, enabling highly precise and customizable sensing elements (Li et al., 2019). Recent advances have extended the application of CRISPR-Cas biosensors to electrochemical platforms and improved detection capabilities for low-concentration analytes (Ma et al., 2023). The potential of the technology extends beyond nucleic acid detection to small molecules, opening up new possibilities for molecular diagnostics (Chen et al., 2022). As research progresses, CRISPR-Cas biosensors promise to revolutionize molecular diagnostics, especially when integrated with microfluidic techniques, which could further improve analytical sensitivity, portability and automation and pave the way for efficient point-of-care diagnostics (Singh et al., 2024). In addition, miniaturization through lab-on-a-chip technologies and microfluidic platforms could facilitate the development of high-throughput portable screening devices for rapid on-site assessment of flavors. Microfluidic lab-on-a-chip (LOC) platforms offer promising solutions for miniaturization and parallelization of biological and chemical assays, enabling high-throughput screening with reduced sample volumes and costs. These platforms offer a range of fluidic units that can be easily combined for different applications, including lateral flow assays, pressure-driven laminar flow and centrifugal microfluidics (Mark et al., 2010). LOC technologies allow up to a million-fold reduction in sample volume and precise spatio-temporal control, enabling highly parallelized assays. Applications range from next-generation sequencing to single-cell studies and high-throughput screening (Vyawahare et al., 2010).

Recent advances in bio-printing technologies offer promising opportunities for the creation of sophisticated three-dimensional cellular architectures that mimic human tissues, including taste buds, which could improve the physiological relevance of biosensor responses. 3D bio-printing allows precise control over the spatial and temporal distribution of cells and extracellular matrix, enabling the fabrication of complex tissue constructs (Arslan-Yildiz et al., 2016). This technology can be integrated into organ-on-chip systems to create more physiologically relevant models for studying cellular interactions and microenvironments (Fetah et al., 2019). In the context of taste sensation research, organoids of taste buds have been successfully cultured and combined with MEAs to develop biosensors that can detect electrophysiological responses to various taste stimuli (Liu et al., 2022). These advances in biofabrication techniques, including bio-printing, have the potential to revolutionize the development of biosensors by improving spatial sensitivity and enabling the creation of more accurate in vitro physiological models (Dias et al., 2014). Such innovations could significantly improve the study of taste perception and the development of novel biosensing applications.

Multi-mode/signal biosensors, especially those incorporating electrochemical techniques, offer improved accuracy, sensitivity and selectivity in the detection of different molecules (Han et al., 2024). Portable bio-electronic sensors that utilize human olfactory and taste receptors have been developed for the simultaneous detection of odour and taste molecules associated with food contamination (Son et al., 2017). These biomimetic sensors, which can be classified into in vitro and in vivo approaches, aim to better understand taste and odour perception through the detection of odorants and tastants (Wu et al., 2017). The integration of biosensors into food quality monitoring systems enables rapid, accurate and on-site detection of contaminants, revolutionizing food safety and quality assurance practices (Nath, 2024). These advances in multimodal biosensor technologies represent a significant step towards more comprehensive and efficient methods of flavor assessment.

Cell immobilization techniques and biomaterial science have shown that they can improve the stability and longevity of cell-based biosensors. Three-dimensional encapsulation of cells using hydrogels has emerged as a potential solution to the limitations of existing 2D cell-based biosensors, offering improved physiological mimicry and detection capability (Zhou et al., 2011). Various immobilization methods including layer-by-layer assembly, covalent bonding and entrapment have been developed to improve the performance of biosensors. The integration of nanomaterials such as graphene and quantum dots into immobilization techniques has the potential to increase the efficacy of biosensors (Pawar & Pawar, 2023). For field applications, strategies such as polymer-based immobilization, microfluidic devices and paper-based systems are being explored (Lobsiger & Stark, 2019). The choice of a suitable immobilization technique is crucial for the development of efficient, simple and cost-effective biosensors with long storage stability and use (Bhardwaj, 2014).

The convergence of artificial intelligence and machine learning algorithms with biosensor data analysis is expected to enhance pattern recognition and prediction capabilities, potentially enabling real-time flavor profiling with unprecedented accuracy. Machine learning algorithms have demonstrated high accuracy in flavor profiling and origin classification of coffee beans (Jang et al., 2024). Various machine learning techniques, including deep learning methods like convolutional and recurrent neural networks, are being applied to enhance biosensor capabilities across multiple domains (Cui et al., 2020). The integration of AI in point-of-care biosensing offers potential for improved diagnostic methodologies, although challenges remain in data security, algorithmic bias, and regulatory compliance (Flynn & Chang, 2024).

## 7 Conclusion

The development of cell-based biosensors has revolutionized the evaluation of food flavors by providing a theoretical basis and demonstrating significant potential. Despite these advances, their widespread application faces major challenges. First, cell sensors require a complex microenvironment. Stem cell-derived organoids offer a solution, but problems such as weak signal expression and the complexity of cultivation remain. It remains uncertain whether artificial cell lines can fully replicate natural cell systems. Second, the limited ability of current cell sensors to support various analyzes requires the discovery of multifunctional cells for broader applications. Third, apoptosis and reduced performance stability hinder long-term storage and consistent functionality. Future research should address these challenges, focusing on optimizing cell culture processes, improving sensor stability, and integrating smart technologies for real-time flavor data acquisition. Combining these efforts with artificial sensory systems can facilitate the adoption of a uniform evaluation method or standard for taste and smell analysis. In addition, the development of portable, multifunctional cell sensors capable of accurately analysing flavors will open up new market opportunities. Advances in cell-based biosensors are not only advancing our understanding of taste and olfactory mechanisms, but also hold transformative potential for food flavor evaluation. As these technologies evolve, they will achieve significant breakthroughs, redefine sensory analysis and drive innovation in food science.

## CRediT authorship contribution statement

**Mahmoud Said Rashed:** Writing – original draft, Visualization, Data curation, Conceptualization. **Esraa A. Abdelkarim:** Writing – original draft, Software, Conceptualization. **Tamer Elsamahy:** Writing – review & editing, Visualization, Formal analysis. **Mabrouk Sobhy:** Writing – original draft, Software, Data curation. **Hany S. El-Mesery:** Supervision, Investigation, Funding acquisition. **Ali Salem:** Investigation, Funding acquisition.

## Declaration of competing interest

The authors declare that they have no known competing financial interests or personal relationships that could have appeared to influence the work reported in this paper.

## Data Availability

Data will be made available on request.
